# Population Admixture and *APOB* Variant Landscape in Ecuadorian Mestizo Patients with Cardiac Diseases: Potential Implications for Familial Hypercholesterolemia Genetics

**DOI:** 10.3390/jcdd13010036

**Published:** 2026-01-08

**Authors:** Santiago Cadena-Ullauri, Patricia Guevara-Ramírez, Viviana A. Ruiz-Pozo, Rafael Tamayo-Trujillo, Elius Paz-Cruz, Manuel Becerra-Fernández, Nieves Doménech, José Luis Laso-Bayas, Rita Ibarra-Castillo, Alejandro Cabrera-Andrade, Ana Karina Zambrano

**Affiliations:** 1 Universidad UTE, Facultad de Ciencias de la Salud Eugenio Espejo, Centro de Investigación Genéticay Genómica, Quito 170129, Ecuador; santiagoa.cadena@ute.edu.ec (S.C.-U.);; 2 EXPRELA Research Group, CICA—Centro Interdisciplinar de Química e Bioloxía, Universidade da Coruña (UDC), 15071 A Coruña, Spain; manuel.becerra@udc.es; 3 Instituto de Investigación Biomédica de A Coruña (INIBIC), As Xubias, 15006 A Coruña, Spain; 4 Instituto de Investigación Biomédica de A Coruña (INIBIC)-CIBERCV, Complexo Hospitalario Universitario de A Coruña (CHUAC), Sergas, Universidade da Coruña (UDC), 15006 A Coruña, Spain; 5 Independent Researcher, Quito 170103, Ecuador; 6 Grupo de Bio-Quimioinformática, Universidad de Las Américas, Quito 170125, Ecuador; raul.cabrera@udla.edu.ec; 7 Carrera de Enfermería, Facultad de Ciencias de la Salud, Universidad de Las Américas, Quito 170125, Ecuador

**Keywords:** next-generation sequencing, *APOB*, Ecuadorian, healthcare, population genetics

## Abstract

Apolipoprotein B (*APOB*) is a key structural component of atherogenic lipoproteins and one of the principal genes implicated in familial hypercholesterolemia (FH). However, *APOB* genetic variation remains poorly characterized in Latin American and admixed populations. In this study, we performed a descriptive analysis of *APOB* variants in 60 Ecuadorian mestizo patients with inherited cardiac conditions using next-generation sequencing (NGS) and genetic ancestry inference. A total of 227 *APOB* variants were identified, the majority of which were classified as benign (n = 220) or likely benign (n = 3) according to ACMG criteria, while three variants were classified as variants of uncertain significance (VUS). The most frequently observed variants included rs1042034, rs679899, rs676210, and rs1367117. Comparative allele-frequency analyses using ALFA and PAGE Latin American reference datasets demonstrated that the *APOB* variant frequencies observed in the cohort were comparable to those reported in other Latin American populations, reflecting the admixed genetic background of Ecuadorian mestizos, predominantly of Native American and European ancestry. No pathogenic *APOB* variants were detected. Although lipid measurements were not available and genotype–phenotype associations could not be assessed, this study provides the first comprehensive overview of *APOB* variation in Ecuadorian mestizo individuals. These findings expand population-specific genomic data for an underrepresented group and underscore the importance of regional reference datasets for accurate variant interpretation in admixed populations.

## 1. Introduction

*APOB* is a critical structural and functional component of atherogenic lipoproteins, including very low-density lipoproteins (VLDL), intermediate-density lipoproteins (IDL), and low-density lipoprotein (LDL) [1]. It serves as the sole apolipoprotein in LDL and functions as the ligand for LDL receptor (LDLR), mediating hepatic clearance of LDL particles [2]. Variants that impair this interaction can disrupt LDL uptake, whereas truncating *APOB* mutations reduce VLDL assembly and may lead to hypocholesterolemia [1,3]. For instance, pathogenic *APOB* variants associated with LDLR-binding defects, such as the well-characterized p.Arg3527Gln mutation, have been described primarily in European and Asian populations [2,4].

Familial hypercholesterolemia (FH) is an inherited disorder of lipid metabolism, characterized by markedly elevated plasma low-density lipoprotein cholesterol (LDL-C) levels and a significantly increased risk of premature atherosclerotic cardiovascular disease (ASCVD) [5,6,7,8]. Pathogenic *APOB* variants represent one of the three primary causes of autosomal dominant FH, alongside mutations in *LDLR* and *PCSK9* [3]. Approximately 5–10% of genetically confirmed FH cases are attributed to *APOB* mutations [5]. However, most *APOB* variants identified to date are benign or of uncertain significance, and their functional impact often remains unknown.

While most studies on FH have focused on identifying single pathogenic variants with large effects, the role of multiple low-impact or benign variants in modifying the disease phenotype is being increasingly recognized [9,10]. In individuals without clearly identifiable pathogenic variants, hypercholesterolemia may result from the cumulative effect of multiple common single nucleotide polymorphisms (SNPs), each exerting a modest influence on LDL-C levels. These polygenic risk scores (PRS) not only provide a potential alternative explanation for hypercholesterolemia but also may serve as phenotype modifiers in those with monogenic FH [11]. Moreover, PRS may help explain the biochemical heterogeneity observed among patients carrying the same monogenic variant, as the presence of multiple common variants can either exacerbate or attenuate the clinical expression of FH [12,13]. Although PRS is not currently considered a diagnostic tool and its utility may vary across populations, it represents a valuable approach for elucidating the genetic architecture and phenotypic variability of hypercholesterolemia [12].

Although FH and *APOB* variants have been studied in populations of European or Asian descent, Latin American populations remain markedly underrepresented in genomic and clinical research [14,15]. This lack of representation limits the interpretation of genetic variants, hinders the development of population-specific reference datasets, and may contribute to disparities in diagnosis, treatment efficacy, and risk assessment [14,15,16]. Furthermore, highly admixed groups, including Ecuadorian Mestizos, which have a mixture of Native American, European, and African contributions, are especially affected, as allele frequencies and variant classifications derived from non-admixed populations may not accurately reflect their genetic architecture [17].

Given that *APOB* variation is understudied in Latin America, and considering the admixed genetic composition of Ecuadorian populations, population-specific allele characterization is essential for accurate genomic interpretation. Thus, this study represents the first comprehensive characterization of *APOB* variants in Ecuadorian Mestizo individuals. The goal of this study is to describe the spectrum of *APOB* variants identified in Ecuadorian mestizo patients with inherited cardiac conditions, by integrating Next-Generation Sequencing with genetic ancestry analysis and describe potential implications for variant interpretation in underrepresented populations.

## 2. Materials and Methods

### 2.1. Study Population

The present study involved the analysis of the *APOB* gene in sixty Ecuadorian mestizo patients diagnosed with an inherited cardiac condition, including hypertrophic cardiomyopathy, long QT syndrome, atrial flutter, atrioventricular block, cardiac arrhythmias, Brugada syndrome, systolic murmur, arrhythmogenic cardiomyopathy, Ebstein anomaly, and Wolff-Parkinson-White syndrome. The ages ranged from 9 days to 70 years old. This cohort included patients derived from the electrophysiological unit from a third-level Ecuadorian hospital (Quito–Ecuador). The individuals must meet the following inclusion criteria: self-reported Ecuadorian mestizo ethnicity and confirmed or suspected hereditary cardiac disorder based on clinical evaluation Lipid profiles, including LDL-C measurements, were not available.

### 2.2. Sampling, DNA Extraction and Quantification

A peripheral blood sample from each individual was collected in EDTA tubes, following the signing of the informed consent. DNA extraction was carried out with the PureLink Genomic DNA Mini Kit, starting from 200 uL of the blood sample according to the manufacturer’s instructions [18]. The DNA quality and quantity was measured by fluorometric method (Qubit) through Broad Range dsDNA Assay (Thermo Fisher Scientific, Waltham, MA, USA) following the manufacturer’s protocol [19].

### 2.3. Next Generation Sequencing

NGS was performed using the TruSight Cardio Kit (Illumina, San Diego, CA, USA), which covers 174 genes associated with 17 inherited cardiac diseases including FH, according to the manufacturer’s protocol [20]. After library preparation, this was diluted and denatured to 10 pM and combined with denatured PhiX (12.5 pM) control to form a final volume of 600 µL, which was loaded into a v2 300 cycle Miseq cartridge, and run at the Illumina Miseq Next Generation Sequencer (Illumina, San Diego, CA, USA) (315 cycles), according to the manufacturer’s instructions [21,22].

### 2.4. Variant Calling and American College of Medical Genetics and Genomics (ACMG) Interpretation

For genomic data, the FASTQ file generated at BaseSpace (Illumina, San Diego, CA, USA) was analyzed in the Dragen Enrichment (v. 3.10.4) software to align it against reference genome sequence (GRCh38), which allowed the identification of genomic variants like SNPs, insertions, deletions, and structural variations (variant calling). After variant calling, the Q30 quality of all variants were analyzed to remove false positives and keep high-confidence variants. Next, the Variant Interpreter (Illumina, San Diego, CA, USA, v2.17) software was used to annotate the *APOB* variants and to convert to the nomenclature recommended by the Human Genome Variation Society (HGVS).

All *APOB* variants were analyzed and classified according to ACMG guidelines [23]. The Franklin^®^ variant interpretation platform (Genoox, Tel Aviv, Israel) was used for automated ACMG evaluation, followed by manual review when discrepancies were detected. Notably, this platform uses information retrieved from clinical databases, including ClinVar [18], and compiles the results of various in silico prediction software programs such as Revel, AlphaMissense, FATHMM, Mut Assesor, SIFT, MutationTaster, DANN, MetaLR, PrimateAI, and BayesDel. Lastly, Franklin^®^ variant interpretation platform also includes the variant frequency based on information of the Genome Aggregation Database (gnomAD v3.1).

### 2.5. Genetic Ancestry Determination

Ancestry Informative Markers (AIMs), which include a collection of insertion/deletion (InDel) polymorphisms was used for ancestry determination, according to the protocol described by Zambrano et al. (2019) [17]. Fragment analysis and capillary electrophoresis were performed using a 3500 Genetic Analyzer from Applied Biosystems (Waltham, MA, USA).

The bioinformatics pipeline included ancestry inference analyses using STRUCTURE software v2.3.4, by comparing genotypic data from the 46 AIMS-InDels polymorphisms against populations of reference reported in the HGDP-CEPH panel (Africans, Europeans, and Native Americans; subset H952) [24,25,26]. An admixture model “Use population information to test for migrants”, with a burn-in length of 10,000, and 10,000 Markov Chain Monte Carlo (MCMC) iterations was used. The number of cluster (K) was evaluated from K = 1 to K = 3, and the results were visualized as triangular plots generated, using STRUCTURE, to illustrate individual ancestry proportions and population admixture patterns.

## 3. Results

### 3.1. Variant Detection in the APOB Gene

**Figure 1:**
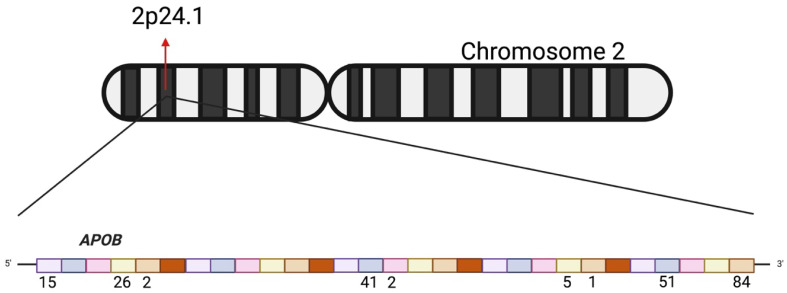
Representation of the *APOB* gene on chromosome 2 (2p24.1). The upper panel shows the chromosomal localization of the gene. The lower panel illustrates the gene structure, where each colored block represents an exon. Numbers below the exons indicate the number of variants identified within each exon in the present study.

This study analyzed genetic variants in 60 Ecuadorian mestizo individuals diagnosed with hereditary cardiovascular disease using the TruSight Cardio Kit (Illumina) for targeted sequencing, resulting in a 20× coverage in 99% of the targeted *APOB* regions. A total of 227 *APOB* gene variants were identified across all samples, some of which were recurrent among different individuals. Figure 1 illustrates the number of variants identified in each exon of the *APOB* gene.

### 3.2. Variant Frequency in the Cohort

The most frequently identified benign variants in the cohort were rs1042034 (c.13013G>A), rs679899 (c.1853C>T), rs1367117 (c.293C>T), and rs13306194 (c.8216C>T). Among these, rs1042034 exhibited the highest frequency, with 54 occurrences across the analyzed samples. This was followed by rs679899, rs13306194, and rs1367117, all of which were recurrently detected in multiple individuals within the study population.

**Figure 2:**
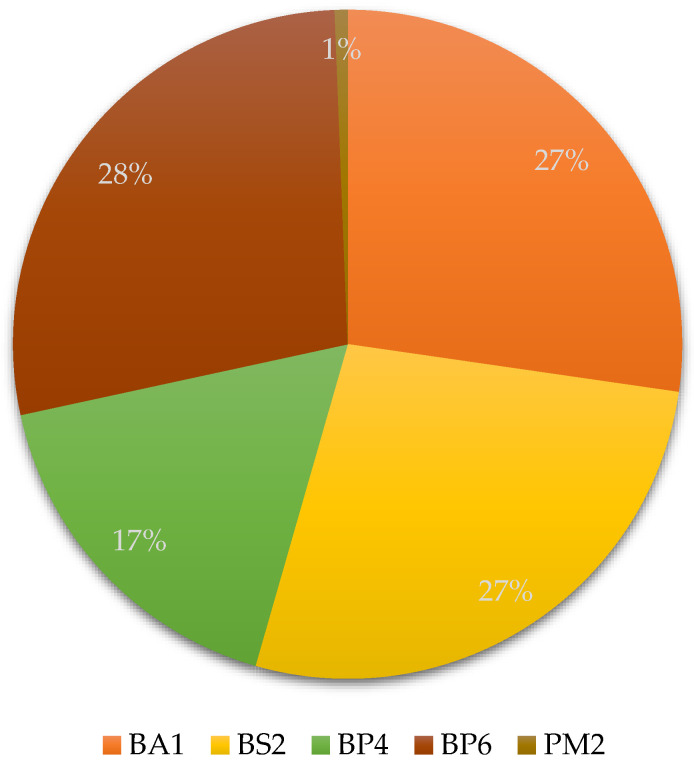
Distribution of *APOB* variants according to ACMG evidence criteria. The pie chart illustrates the proportion of *APOB* gene variants identified in the study. Most variants were categorized as benign based on population frequency and computational data: BA1 (benign stand-alone), BS2 (benign strong), BP4 and BP6 (benign supporting). Only a minor fraction of variants exhibited PM2 (pathogenic moderate) evidence, indicating very low population frequency but insufficient data for definitive pathogenic classification.

A total of 227 genetic variants were identified in the *APOB* gene. Of these, 220 were classified as benign, 3 as likely benign, and 4 as variants of uncertain significance (VUS) according to the ACMG guidelines (Figure 2). In addition to common variants, several low-frequency and benign variants were also identified in the *APOB* gene, each detected in a single individual. Among the benign variants, rs533617 (c.5768A>G), rs12713843 (c.3383G>A), rs72653077 (c.3427C>T), rs61736761 (c.3634C>A), and rs12713450 (c.13451C>T) were observed. Furthermore, three variants were classified as likely benign rs141225768 (c.4663A>G), rs72653098 (c.8912A>C), and rs142638069 (c.5110G>A). Additionally, three variants were categorized as VUS such as rs531341535 (c.3443T>A), rs539614975 (c.3379C>T), and rs769491475 (c.9871C>T). The detection of these rare variants emphasizes the genetic heterogeneity present in the Ecuadorian mestizo population. The use of a cardiovascular genetic panel enabled the detection of common and rare variants associated with familial hypercholesterolemia in the *APOB* gene in Ecuadorian Mestizo population.

### 3.3. Comparative Allele Frequencies with Population Databases

In addition to the variants classification, Table 1 lists all *APOB* variants identified in the cohort, including HGVS annotations, molecular consequences, zygosity, ACMG classifications with associated criteria. For each variant, the number of carriers and the corresponding cohort frequency are reported, providing a comprehensive overview of *APOB* genetic variation in this population.

**Figure 3:**
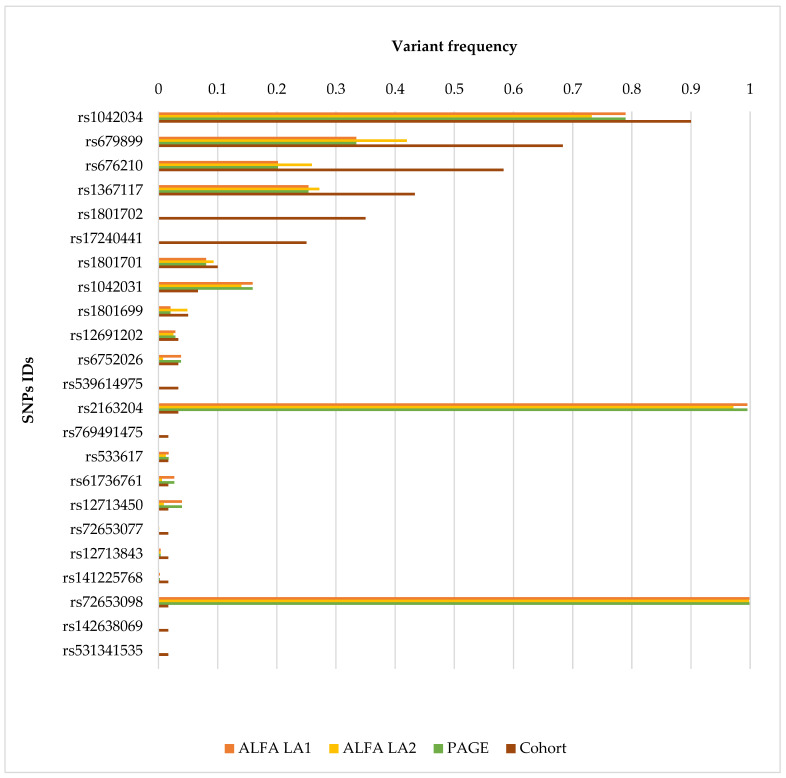
Comparison of *APOB* Variant Allele Frequencies in Ecuadorian Patients and Reference Populations. The bar chart compares the allele frequencies of *APOB* gene variants identified in a cohort of Ecuadorian patients with suspected hereditary cardiac conditions against reference populations. Each bar corresponds to a specific single nucleotide polymorphism (SNP), identified by its SNP ID on the y-axis. The x-axis represents allele frequency values ranging from 0 to 1. Four data sources are shown per variant: ALFA Latin American 1 (LA1) (orange), ALFA Latin American 2 (LA2) (blue), PAGE database (green), and the study cohort (brown). This graphical representation allows for a direct visual comparison of variant frequencies between the Ecuadorian cohort and Latin American populations reported in public databases.

Figure 3 compares the allele frequencies of *APOB* gene variants identified in our Ecuadorian patient cohort with those reported in ALFA, and PAGE population databases. Furthermore, Supplementary Table S1 summarizes missense variants identified in the cohort, indicating SNP identifiers, HGVS nomenclature, predicted consequences, ACMG classification and criteria, population allele frequencies (ALFA and PAGE), the number of carriers, and the corresponding variant frequencies within the study cohort.

### 3.4. Genetic Ancestry Determination

**Figure 4:**
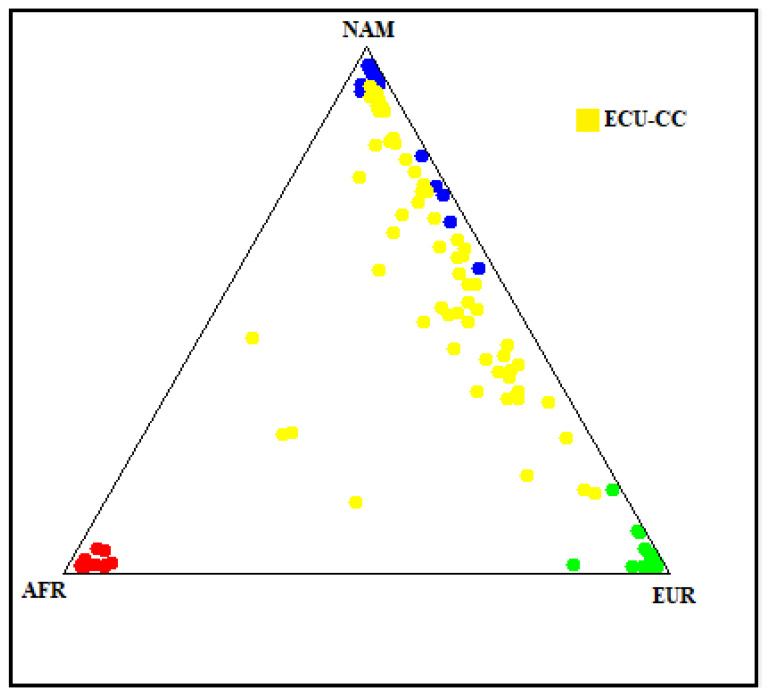
Genetic ancestry composition of the Ecuadorian Mestizo cohort. The plot illustrates the genetic ancestry proportions of the study cohort (yellow dots, labeled ECU-CC) in comparison with reference populations of African (AFR, red), Native American (NAM, blue), and European (EUR, green) origin. Each point represents an individual’s ancestry component estimated from 46 AIMs polymorphisms.

Ancestry analysis of the Ecuadorian Mestizo cohort showed a mainly Native American genetic background (58.3%), followed by European (32.9%) and African (8.8%) components. Figure 4 illustrates that most individuals cluster along the Native American and European axis, with a minimal dispersion toward the African vertex. This pattern is consistent with the admixed genetic composition historically described in the Ecuadorian populations. The predominance of Native American ancestry could provide crucial information for interpreting allele frequency differences and may identify potential population-specific variants observed in the *APOB* gene.

## 4. Discussion

*APOB* mutations account for approximately 5–10% of all FH cases, which could potentially lead to a compromised cardiovascular health due to elevated levels of LDL-C [27]. These mutations typically occur within the LDL receptor-binding domain of apolipoprotein B-100, disrupting receptor interaction and leading to the accumulation of LDL-C in plasma [1,28]. However, many variants in *APOB* are located outside this functional region or have not been experimentally characterized, limiting their clinical interpretation [20,27,28].

A comprehensive study that compiled global *APOB* variant data up to November 2015 identified 97 distinct variants associated with FH. Of these, 83% were missense and 97% were single-nucleotide substitutions. Importantly, 80.4% lacked sufficient functional evidence and were classified as variants of uncertain significance (VUS) based on ACMG criteria. The geographical distribution of these variants was markedly skewed, with countries such as Portugal (40 variants), France (12), and Germany (9) reporting high mutational diversity, while no pathogenic *APOB* variants were documented in African populations. These findings underscore the underrepresentation of many regions, including Latin America, in genomic studies and highlight the need of population-specific research [20].

Ethnic and regional variability in *APOB* variation has been described in several populations. For instance, the Malaysian Health and Wellbeing Assessment reported frequencies of *APOB* pathogenic variants comparable to *LDLR* mutations [29], while other studies highlight inconsistencies in variant annotation and classification across databases [29,30]. These disparities further complicate variant interpretation, especially in admixed populations.

The ancestry composition of the patients included in the study provides noteworthy context for interpreting the *APOB* variants identified. The predominance of Native American ancestry (58.3%), together with European (32.9%) and African (8.8%) proportions, reflects the characteristic admixture of the Ecuadorian Mestizo population, which aligns with previously reported studies [17].

Consistent with global datasets, most *APOB* variants detected in our study were classified as benign according to ACMG criteria. The absence of pathogenic variants may be attributed to our cohort not being selected for hypercholesterolemia and the unavailability of lipid measurements (including LDL-C), preventing a direct genotype–phenotype correlation. Nonetheless, several recurrent benign variants identified in our cohort have been associated with lipid traits or cardiometabolic phenotypes in other populations. Examples include the rs2163204 (c.8353A>C) variant, which has been identified in risk haplotypes associated with hyperlipidemia and exists in linkage disequilibrium with other functional variants of the *APOB* gene (rs1042034, rs676210, and rs679899) [31]. Furthermore, thers1801702 (c.12809G>C) variant has also shown significant associations with elevated total cholesterol and LDL-C levels in Latin American populations. Specifically, individuals of Mayan ancestry carrying the G allele demonstrated higher lipid concentrations [32], a finding replicated in Caribbean Hispanics in Manhattan [33]. Although this variant has been classified as benign under monogenic disease criteria, its cumulative effect suggests an important functional role within the spectrum of polygenic hypercholesterolemia, especially in underrepresented population contexts [33,34].

Similar cases include the rs679899 variant, which population-level associations have suggested broader implications in LDL-C levels and chronic kidney disease in hypertensive patients [35,36,37,38]. More examples include the rs1042031 [34,39,40,41,42,43,44], rs533617 [45,46,47,48] rs17240441 [30,48,49,50], rs61736761 [17,51,52], rs1042034 [53,54,55,56], rs1367117 [57,58,59], rs6752026 [4,36,60,61,62,63,64], and rs676210 [65,66,67,68]. Although these associations are heterogeneous and often population-specific, they collectively illustrate that *APOB* variation may modulate lipid phenotypes within polygenic or context-dependent models. However, such mechanisms cannot be evaluated in the present study due to the lack of available lipid data and the heterogeneous clinical indications of the cohort.

Lastly, among the variants identified in our cohort, nine more missense variants were also identified: rs141225768, rs72653098, rs72653077, rs127113450, rs1801699, rs1801701, rs12713843, rs142638069, and rs12691202. According to ACMG criteria, these variants have been classified as benign, and likely benign; however, there is no evidence of the potential pathogenicity of the variants, as these substitutions may alter structural domains and could collectively influence the lipid phenotypes. Thus, further functional characterization is required to understand the impact of these changes.

Therefore, the characterization of benign variants should not be overlooked in the context of FH. Especially in underexplored populations, such as the Ecuadorian mestizo population, because these types of variants could exert a cumulative effect that contributes to the hyperlipidemic phenotype, even in the absence of classic pathogenic mutations. This context encourages us to rethink the functional significance of these variants within models of polygenic inheritance and underscores the necessity to adjust clinical and genetic algorithms to reflect more diverse and multifaceted situations [11,34,69].

Moreover, this scenario highlights the need for implementing more advanced genetic characterization strategies, including cascade testing, in silico prediction tools, and experimental validation [11]. By incorporating variants typically considered benign, which may have functional significance in complex inheritance models, this approach can significantly enhance the stratification of cardiovascular risk.

Notably, our study identified three VUS: rs539614975, rs769491475, and rs531341535. These subset of variants exhibit low allele frequencies across reference datasets, which could suggest their relevance as rare variants and potential clinical implications. The absence of these variants at high frequencies in healthy populations supports the hypothesis that they could be of pathogenic significance. Furthermore, the overall similarity between the cohort and ALFA/PAGE allele frequencies highlights the genetic representativeness of our study population within the broader Latin American context. These findings reinforce the utility of population-specific databases for interpreting genetic variants in admixed populations.

Although benign variants are traditionally assumed to have minimal functional impact, accumulating evidence suggests that multiple low-effect variants can collectively influence lipid traits and may contribute to polygenic hypercholesterolemia [70,71,72,73,74]. A variant deemed benign in one population may potentially contribute to disease susceptibility in specific genetic contexts. Furthermore, the accumulation of multiple benign variants, each with minimal effect on its own, can collectively reach a threshold where the combined genetic burden influences disease risk [73,74]. Given the admixed ancestry of Ecuadorians, the cumulative burden of benign variants may differ from that of other populations. However, without LDL-C data, this possibility remains hypothetical and should be addressed in future studies integrating polygenic scores, lipid measurements, and functional assays.

The present study has limitations. First, LDL-C and other lipid parameters were unavailable, preventing direct phenotype–genotype analyses and limiting clinical interpretation. Second, patients were not selected based on hypercholesterolemia, but rather on diverse hereditary cardiac disorders, which restricts comparisons to FH-oriented studies. Furthermore, the study lies in the lack of in vitro or in vivo functional analyses that would confirm the biological relevance of the identified benign variants, particularly those with indirect or contextual associations with lipid metabolism. Additionally, the clinical interpretation of variants in highly polymorphic genes such as *APOB* may be limited by the limited availability of representative databases for Latin American populations, which could have influenced the automated classification of some variants according to the ACMG criteria.

Lastly, the limited sample size, and the absence of segregation analyses and a healthy control group hinder the precise assessment of the statistical significance of allele frequencies within a larger epidemiological framework. Future research should consider integrating polygenic, transcriptomic, and epigenetic analyses, as well as cellular models, to assess the functional impact of the variants.

In conclusion, this study provides the first descriptive analysis of *APOB* variation in Ecuadorian mestizo individuals using next-generation sequencing and ancestry inference. Although no pathogenic *APOB* variants were identified, the dataset expands regional genomic knowledge and highlights the importance of population-specific allele frequencies for variant interpretation. The identification of rare VUS and the predominance of benign variants underscore the need for broader genomic research in Latin America, including functional and clinical studies, to better understand the implications of *APOB* variation in genetically admixed populations.

## Figures and Tables

**Table 1:** Missense variants identified in the APOB gene in the study cohort.

SNP ID	HGVSC	HGVSP	Consequence	Suggested ACMG Classification	ACMG Criteria	Number of Individuals	Cohort MAF
rs1042034	c.13013G>A	p.(Ser4338Asn)	missense	Benign	BA1, BS2, BP4, BP6	54	0.9000
			variant				
rs679899	c.1853C>T	p.(Ala618Val)	missense	Benign	BA1, BS2, BP6	41	0.6833
			variant				
rs676210	c.8216C>T	p.(Pro2739Leu)	missense	Benign	BA1, BS2, BP6	35	0.5833
			variant				
rs1367117	c.293C>T	p.(Thr98Ile)	missense	Benign	BA1, BS2, BP4, BP6	26	0.4333
			variant				
rs1801702	c.12809G>C	p.(Arg4270Thr)	missense	Benign	BA1, BS2, BP4, BP6	21	0.3500
			variant				
rs17240441	c.35_43delTGGCGCTGC	p.(Leu12_Leu14del)	missense	Benign	BA1, BS2, BP3, BP6	15	0.2500
			variant				
rs1801701	c.10913G>A	p.(Arg3638Gln)	missense	Benign	BA1, BS2, BP4, BP6	6	0.1000
			variant				
rs1042031	c.12541G>A	p.(Glu4181Lys)	missense	Benign	BA1, BS2, BP4, BP6	4	0.0667
			variant				
rs1801699	c.5741A>G	p.(Asn1914Ser)	missense	Benign	BA1, BS2, BP6	3	0.0500
			variant				
rs12691202	c.2188G>A	p.(Val730Ile)	missense	Benign	BA1, BS2, BP4, BP6	2	0.0333
			variant				
rs6752026	c.433C>T	p.(Pro145Ser)	missense	Benign	BA1, BS2, BP6	2	0.0333
			variant				
rs539614975	c.3379C>T	p.(Pro1127Ser)	missense	VUS	PM2	2	0.0333
			variant				
rs2163204	c.8353A>C	p.(Asn2785His)	missense	Benign	BA1, BS2, BP4, BP6	2	0.0333
			variant				
rs769491475	c.9871C>T	p.(Arg3291Cys)	missense	VUS	PM2, BP6	1	0.0167
			variant				
rs533617	c.5768A>G	p.(His1923Arg)	missense	Benign	BA1, BS2, BP6	1	0.0167
			variant				
rs61736761	c.3634C>A	p.(Leu1212Met)	missense	Benign	BA1, BS2, BP6	1	0.0167
			variant				
rs12713450	c.13451C>T	p.(Thr4484Met)	missense	Benign	BA1, BS2, BP4, BP6	1	0.0167
			variant				
rs72653077	c.3427C>T	p.(Pro1143Ser)	missense	Benign	BA1, BS2, BP4, BP6	1	0.0167
			variant				
rs12713843	c.3383G>A	p.(Arg1128His)	missense	Benign	BS1, BS2, BP6	1	0.0167
			variant				
rs141225768	c.4663A>G	p.(Ile1555Val)	missense	Likely Benign	BS1, BP4, BP6	1	0.0167
			variant				
rs72653098	c.8912A>C	p.(Asn2971Thr)	missense	Likely Benign	PM2, BP4, BP6	1	0.0167
			variant				
rs142638069	c.5110G>A	p.(Ala1704Thr)	missense	Likely benign	BP4, BP6	1	0.0167
			variant				
rs531341535	c.3443T>A	p.(Leu1148His)	missense	VUS	PM2, BP6	1	0.0167
			variant				

## Data Availability

Variants have been presents in the article. However, due to the size of the information and the fact that some information will be part of other articles, data may be made available upon a considerable request.

## Supplementary Materials

The following supporting information can be downloaded at: https://www.mdpi.com/article/10.3390/jcdd13010036/s1: Supplementary Table S1: Missense Variants Identified in the Cohort.
