# Supplementary material for: Population Admixture and APOB Variant Landscape in Ecuadorian Mestizo Patients with Cardiac Diseases: Potential Implications for Familial Hypercholesterolemia Genetics
Source: J Cardiovasc Dev Dis. 2026 Jan 8;13(1):36. doi: 10.3390/jcdd13010036 (PMC12841957; doi:10.3390/jcdd13010036)
Supplement: Supplementary file 1 [file jcdd-13-00036-s001.zip › jcdd-4008996-supplementary.pdf]

Journal of
Cardiovascular Development and Disease

MDPI

Supplementary Materials

Supplementary Table S1. Missense Variants Identified in the Cohort

SNP ID	HGVSC	HGVSP	Consequence	Suggested ACMG classification	ACMG criteria	ALFA	PAGE	Number of individuals	Variant frequency in the cohort
rs1042034	c.13013G>A	p.(Ser4338Asn)	missense variant	Benign	BA1, BS2, BP4, BP6	LA1 0,7893<br>LA2 0,73226	SA 0,7255	54	0,9000
rs679899	c.1853C>T	p.(Ala618Val)	missense variant	Benign	BA1, BS2, BP6	LA1 0,3342<br>LA2 0,41981	SA 0,3794	41	0,6833
rs676210	c.8216C>T	p.(Pro2739Leu)	missense variant	Benign	BA1, BS2, BP6	LA1 0,2018<br>LA2 0,25946	SA 0,2714	35	0,5833
rs1367117	c.293C>T	p.(Thr98Ile)	missense variant	Benign	BA1, BS2, BP4, BP6	LA1 0,2535<br>LA2 0,27	SA 0,2714	26	0,4333
rs1801702	c.12809G>C	p.(Arg4270Thr)	missense variant	Benign	BA1, BS2, BP4, BP6	LA1 0<br>LA2 0	SA Not Reported	21	0,3500
rs17240441	c.35_43del-TGGCGCTGC	p.(Leu12_Leu14 del)	missense variant	Benign	BA1, BS2, BP3, BP6	LA1 0<br>LA2 0	SA Not Reported	15	0,2500
rs1801701	c.10913G>A	p.(Arg3638Gln)	missense variant	Benign	BA1, BS2, BP4, BP6	LA1 0,0805<br>LA2 0,09295	SA 0,0933	6	0,1000
rs1042031	c.12541G>A	p.(Glu4181Lys)	missense variant	Benign	BA1, BS2, BP4, BP6	LA1 0,1590<br>LA2 0,1401	SA 0,11	4	0,0667
rs1801699	c.5741A>G	p.(Asn1914Ser)	missense variant	Benign	BA1,BS2, BP6	LA1 0,0203<br>LA2 0,0489	SA 0,9506	3	0,0500
rs12691202	c.2188G>A	p.(Val730Ile)	missense variant	Benign	BA1, BS2, BP4, BP6	LA1 0,0285<br>LA2 0,0253	SA Not Reported	2	0,0333
rs6752026	c.433C>T	p.(Pro145Ser)	missense variant	Benign	BA1, BS2, BP6	LA1 0,0382<br>LA2 0,0076	SA 0,0106	2	0,0333
rs539614975	c.3379C>T	p.(Pro1127Ser)	missense variant	VUS	PM2	LA1 Not Reported<br>LA2 Not Reported	SA Not Reported	2	0,0333
rs2163204	c.8353A>C	p.(Asn2785His)	missense variant	Benign	BA1,BS2, BP4, BP6	LA1 0,9952<br>LA2 0,9717	SA 0,9818	2	0,0333
rs769491475	c.9871C>T	p.(Arg3291Cys)	missense variant	VUS	PM2, BP6	LA1 0<br>LA2 0	SA Not Reported	1	0,0167
rs533617	c.5768A>G	p.(His1923Arg)	missense variant	Benign	BA1, BS2, BP6	LA1 0,0172<br>LA2 0,01221	SA 0,0096	1	0,0167
rs61736761	c.3634C>A	p.(Leu1212Met)	missense variant	Benign	BA1, BS2, BP6	LA1 0,0268<br>LA2 0,0058	SA 0,0056	1	0,0167
rs12713450	c.13451C>T	p.(Thr4484Met)	missense variant	Benign	BA1, BS2, BP4, BP6	LA1 0,0397<br>LA2 0,0088	SA 0,0151	1	0,0167
rs72653077	c.3427C>T	p.(Pro1143Ser)	missense variant	Benign	BA1, BS2, BP4, BP6	LA1 0,0009<br>LA2 0,0012	SA 0,0005	1	0,0167
rs12713843	c.3383G>A	p.(Arg1128His)	missense variant	Benign	BS1, BS2, BP6	LA1 0,0037<br>LA2 0,00379	SA 0,004	1	0,0167
rs141225768	c.4663A>G	p.(Ile1555Val)	missense variant	Likely Benign	BS1, BP4, BP6	LA1 0,0024<br>LA2 0	SA 0	1	0,0167
rs72653098	c.8912A>C	p.(Asn2971Thr)	missense variant	Likely Benign	PM2, BP4, BP6	LA1 0,9986<br>LA2 0,9979	SA 0,9995	1	0,0167
rs142638069	c.5110G>A	p.(Ala1704Thr)	missense variant	Likely benign	BP4, BP6	LA1 0<br>LA2 0,0004	SA 0	1	0,0167
rs531341535	c.3443T>A	p.(Leu1148His)	missense variant	VUS	PM2, BP6	LA1 0<br>LA2 0	SA Not Reported	1	0,0167

https://doi.org/10.3390/jcdd13010036
